# Development of a 3D Parallel Mechanism Robot Arm with Three Vertical-Axial Pneumatic Actuators Combined with a Stereo Vision System

**DOI:** 10.3390/s111211476

**Published:** 2011-12-05

**Authors:** Mao-Hsiung Chiang, Hao-Ting Lin

**Affiliations:** Department of Engineering Science and Ocean Engineering, National Taiwan University, No.1, Sec.4, Roosevelt Rd., Taipei 106, Taiwan; E-Mail: d97525009@ntu.edu.tw

**Keywords:** sensor collaboration, parallel mechanism robot, pneumatic actuator, kinematics analysis, path tracking control, stereo vision

## Abstract

This study aimed to develop a novel 3D parallel mechanism robot driven by three vertical-axial pneumatic actuators with a stereo vision system for path tracking control. The mechanical system and the control system are the primary novel parts for developing a 3D parallel mechanism robot. In the mechanical system, a 3D parallel mechanism robot contains three serial chains, a fixed base, a movable platform and a pneumatic servo system. The parallel mechanism are designed and analyzed first for realizing a 3D motion in the X-Y-Z coordinate system of the robot’s end-effector. The inverse kinematics and the forward kinematics of the parallel mechanism robot are investigated by using the Denavit-Hartenberg notation (D-H notation) coordinate system. The pneumatic actuators in the three vertical motion axes are modeled. In the control system, the Fourier series-based adaptive sliding-mode controller with H_∞_ tracking performance is used to design the path tracking controllers of the three vertical servo pneumatic actuators for realizing 3D path tracking control of the end-effector. Three optical linear scales are used to measure the position of the three pneumatic actuators. The 3D position of the end-effector is then calculated from the measuring position of the three pneumatic actuators by means of the kinematics. However, the calculated 3D position of the end-effector cannot consider the manufacturing and assembly tolerance of the joints and the parallel mechanism so that errors between the actual position and the calculated 3D position of the end-effector exist. In order to improve this situation, sensor collaboration is developed in this paper. A stereo vision system is used to collaborate with the three position sensors of the pneumatic actuators. The stereo vision system combining two CCD serves to measure the actual 3D position of the end-effector and calibrate the error between the actual and the calculated 3D position of the end-effector. Furthermore, to verify the feasibility of the proposed parallel mechanism robot driven by three vertical pneumatic servo actuators, a full-scale test rig of the proposed parallel mechanism pneumatic robot is set up. Thus, simulations and experiments for different complex 3D motion profiles of the robot end-effector can be successfully achieved. The desired, the actual and the calculated 3D position of the end-effector can be compared in the complex 3D motion control.

## 1. Introduction

Industrial robots are widely used in automobile, mechanical, semiconductor, electronic, and food and beverage industries, and have gradually replaced the labor force [1]. Industrial robots consist of serial type robots and parallel type robots. Serial type robots consist of links sequentially connected to form an open chain with high flexibility and is able to be operated on a larger scope. However, serial type robots have some intrinsic disadvantages such as lower position accuracy affected by the error superposition of each joint and link, slower response due to the series mechanism and poor stiffness for handling heavier loads. On the contrary, parallel type robots which have the end-effector connected to a fixed base by multiple kinematic chains have high ratios of rigidity to weight, high stiffness, high accuracy, high response and good ability to carry heavier loads. The main drawbacks of the parallel type robots lie in a smaller workspace because of their mechanism design, complex kinematics analysis, and the closed-mechanism [2–5]. In recent years, high response, high accuracy and good stiffness are in demand for robots in many applications so parallel type robots have become more popular in industrial automation due to their serial type robot advantages, *i.e.*, high stiffness, high motion accuracy and high load-structure ratio [6,7]. Moreover, the position of the end-effector of robot is calculated by the position sensors of the actuators through kinematic analysis, but the calculated 3D position of the end-effector cannot consider the manufacturing and assembly tolerance of the joints and the parallel mechanism so that errors exist between the actual position and the calculated 3D position of the end-effector, thus influencing the position accuracy of the robot end-effector [8].

Pneumatic actuators have been in use since the 1950s and are widely used in industries as automation systems which emphasize reliability, cost, cleanness, simplicity, easy maintenance and safety in operation. In recent years, the accessibility of low-cost microprocessors and pneumatic components has made possible the use of more complicated control methods in servo pneumatic control systems. Many researchers, therefore, have started using pneumatic actuators such as servo-controlled pneumatic systems [9–12] to work on more complicated motion control tasks and quite suitable to be applied in robotic fields [13–15]. Although a pneumatic actuator is inexpensive and simple, when compared with electro-mechanical actuators with identical power, it is still not competitive in a few applications that demand accuracy, versatility, and flexibility. This is due to some inherent disadvantages of a pneumatic actuator including high nonlinearity, low natural frequency resulting from low stiffness of the air compressibility, and complexity in control because of low damping associated with nonlinearities, time-varying effects, and position dependency. As a result, the pneumatic servo system is a highly nonlinear system and does not easily yield accurate mathematic models [16]. The result is the difficulty and complication in detecting the pneumatic servo control.

Research in the field of pneumatic servo control has been carried out since the 1960s. Some control algorithms like PID, state-space and adaptive control in pneumatic servo systems were developed via higher speed microcomputers in the 1980s. In recent years, due to the development of modern control theories, the problems of the pneumatic servo control have gradually been solved [17–20]. The sliding-mode control (SMC) method has been adopted to handle system nonlinear behaviors, model uncertainty, and bounded disturbances [21,22]. However, the conventional SMC method is model-based and hence is dictated by a varying system model and uncertain parameter values of the system in designing a controller. To deal with these uncertainty problems, Fourier’s series-based functional approximation techniques have been used. Huang *et al.* [23] have suggested an adaptive sliding controller using a Fourier series-based functional approximation technique to handle a nonlinear system containing time-varying, uncertain parameters. Tsai and Huang [24] proposed a FAT-based adaptive controller for pneumatic servo systems with variable payload and uncertain disturbances. Chiang *et al.* [25] proposed a Fourier series-based adaptive sliding-mode controller with *H*_∞_ tracking performance (FSB-ASMC+*H*_∞_) for the rod-less pneumatic cylinder system. The proposed method can not only be effective in preventing approximation errors, disturbances, and un-modeled dynamics, but it also guarantees a desired *H*_∞_ tracking performance for the overall system.

The aim of the present paper was to develop and implement a novel 3D parallel mechanism robot with three vertical-axial pneumatic actuators for path tracking servo control. The 3D robot contains serial manipulators, parallel links, base plates, a movable platform and three vertical pneumatic actuators controlled by pneumatic servo valves. The parallel mechanism is designed and analyzed first for realizing a 3D motion in the X-Y-Z coordinate system of the robot’s end-effector. The inverse kinematics and the forward kinematics of the parallel mechanism robot are investigated using the Denavit-Hartenberg notation (D-H notation) coordinate system. Three optical linear scales are used to measure the position of the three pneumatic actuators. The 3D position coordinates of the end-effector are then calculated from the measuring position of the three pneumatic actuators by means of kinematics. However, the calculated 3D position coordinate of the end-effector is influenced by the manufacturing and assembly tolerance of joints and parallel mechanism, so measuring errors exist between the actual and the calculated 3D position coordinates of the end-effector. To improve this situation, sensor collaboration is developed in this paper. A stereo vision system is used to collaborate with the three position sensors of pneumatic actuators. The stereo vision system combining two CCDs and developed in the authors’ previous publication [8] serves to measure the actual 3D position of the end-effector and calibrate the error between the actual and the calculated 3D position of the end-effector. For controller design, the pneumatic actuators in the three vertical motion axes are modeled. In order to realize 3D path tracking control accuracy of the end-effector, the Fourier series-based adaptive sliding mode controller with *H_∞_* tracking performance is used for the path tracking control of the three vertical pneumatic servo actuators. Moreover, in order to confirm the feasibility of the developed parallel mechanism robot driven by three vertical pneumatic servo actuators, a full-scale test rig of parallel mechanism pneumatic robot is set up. Thus, simulations and practical experiments for different complex 3D motion profiles of the robot end-effector can be successfully implemented. The desired, the actual and the calculated 3D position variations of the end-effector are also compared and calibrated in the complex 3D motion control through the sensor collaboration.

## 2. Test Rig Layout

**Figure 1.**
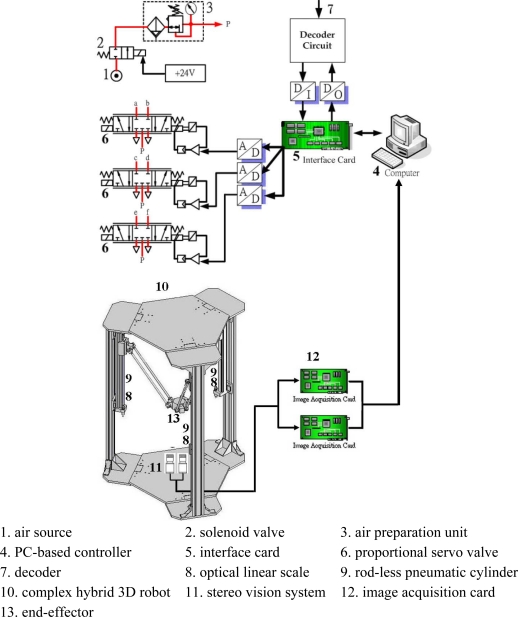
Test rig layout of 3D parallel mechanism robot arm with three vertical-axial pneumatic actuators and a stereo vision system.

In order to verify the proposed parallel mechanism robot driven by three vertical pneumatic servo actuators experimentally, the test rig is set up, whose layout is shown in Figure 1.

**Figure 2.**
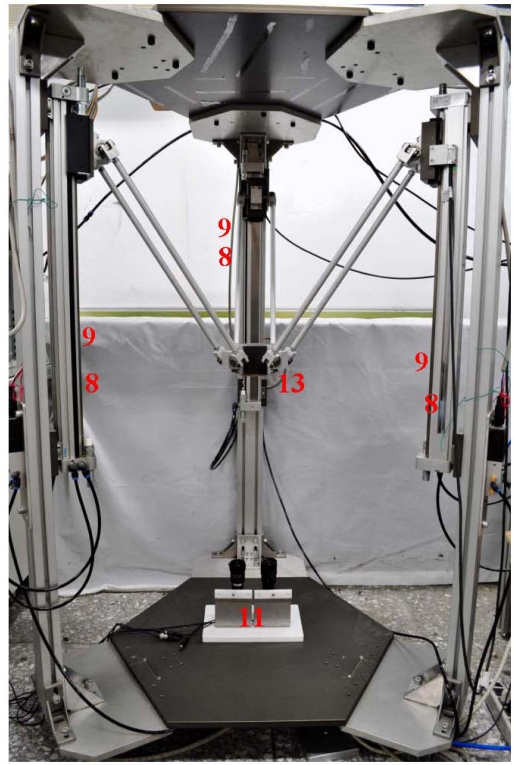
Prototype of the test rig.

A photograph is shown in Figure 2. Table 1 shows the specifications of the test rig. The complex 3D parallel mechanism robot mainly comprises the parallel mechanism and the PC-based control unit. The parallel mechanism consists of a three-axial parallel mechanism with three vertical pneumatic axes arranged at 120° to each other and connected with parallel links for achieving a three-dimensional motion of the end-effector.

The pneumatic servo actuators contain a pneumatic source, three rod-less pneumatic actuators, and three proportional servo valves. The rod-less pneumatic actuator with a piston diameter of 25 mm and a stroke of 500 mm was used for three vertical axes. The proportional servo valve was made by FESTO AG with model MPYE-5-M5-010-B for each vertical axis. The air pressure is set as 5 bars.

In the PC-based control unit using experimental software, and three optical linear scales, an AD/DA interface card and a counter card are included. The control signals are computed in the PC-based control unit and given to the proportional servo valves via an AD/DA interface card with the sampling frequency of 900 Hz in order to drive the vertical rod-less pneumatic actuators. Thus, the end-effector can move in the three-dimensional motion via parallel links driven by the three rod-less pneumatic actuators. In addition, three optical linear scales with resolution of 0.1 μm are necessary for measuring the position of each vertical pneumatic actuator. The measuring position signals of the optical linear scales of the three pneumatic actuators are fed back to PC-based control unit via a counter card. In addition, the control algorithm that will be discussed in Section 3.2 is computed by a 32-bit Open Watcom C language program. Besides, for realizing sensor collaboration the stereo vision system is used in this study, as shown in Figure 1, which is composed of two identical CCD cameras equipped with camera lenses, and the baseline distance is 7.2 cm. The end-effector is the desired target of the stereo vision measurement system, so the actual position of the end-effector can be measured by the stereo vision system. The detailed specifications of the camera are shown in Table 1.

## 3. System Analysis

In this paper, the complex 3D parallel mechanism robot with three pneumatic servo actuators is implemented for 3D path tracking control. First, the 3D path profiles of the end-effector are planned, and next the path profiles of the three pneumatic servo actuators are calculated through the inverse kinematics. The three vertical rodless pneumatic cylinders can then be controlled via the path controllers and through the parallel mechanism to achieve a 3D motion of the end-effector with high accuracy, high stiffness and high response. Furthermore, the end-effector is fitted in a movable platform and connected to the three vertical pneumatic cylinders via three parallel mechanisms. Each chain contains a link and joints driven by a rodless pneumatic cylinder. In this section, the kinematic analysis of the complex 3D parallel mechanism robot, dynamic models of the pneumatic servo system and the controller design are introduced.

### 3.1. Kinematics Analysis

**Figure 3.**
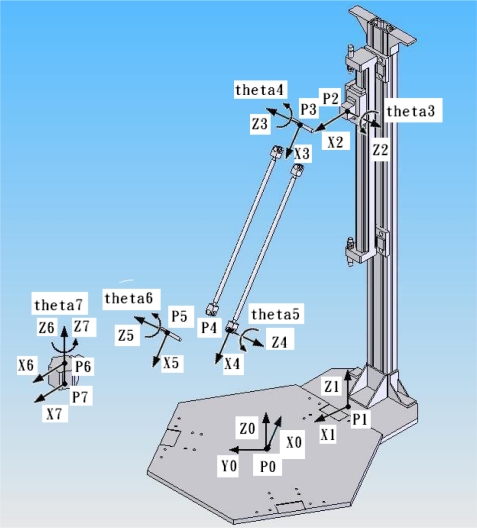
The coordinate frames of the complex hybrid 3D robot with parallel links via nonlinear pneumatic servo system.

The complex 3D parallel mechanism robot contains a movable platform with end-effector connected to a base plate through three parallel kinematic chains driven by the three vertical pneumatic actuators. The motion of the movable platform is transmitted by links and joints. In order to analyze the kinematic model of the 3D parallel mechanism robot, the Denavit-Hartenberg notation (D-H notation) [26] is used to resolve the geometric relation and derive the inverse and forward kinematics. Figure 3 schematically depicts the coordinate frames of the complex 3D parallel mechanism robot system, where P0 to P7 denote the different coordinate frames.

#### 3.1.1. Inverse Kinematics

The inverse kinematics analysis is to solve all possible sets of the positions and the joint angles in a Cartesian space of the three vertical pneumatic linear actuators from a given path position of the end-effector of the moveable platform. In order to analyze the inverse kinematics, the D-H notation method is used for solving the relationship of the elements of the 3D parallel mechanism robot. The D-H notation transformation is described by the following four parameters:

*a_i_* : distance from *Ẑ_i_* to *Ẑ*_*i*+1_ measured along *X̂_i_*
*α_i_* : angle from *Ẑ_i_* to *Ẑ*_*i*+1_ measured about *X̂_i_*
*d_i_* : distance from *X̂*_*i*–1_ to *X̂_i_* measured along *Ẑ_i_*
*θ_i_* : angle from *X̂*_*i*–1_ to *X̂_i_* measured about *Ẑ_i_*

Then, the link transformations can be multiplied together to find the single transformation that relates frame {*N*} to frame {0}:

(1) TN0=T10T21T32…TNN−1

where TN0 is a function of all *n* joint variables and the Cartesian pose of the last link can be computed.

Table 2 shows the link parameters of the complex 3D parallel mechanism robot. As a result, let P7 be the end-effector located on the moving platform, and then the mathematic models of the inverse kinematics have three chains kinematics as follows:

(2) A chain kinematics: (T70)A=(T10)A(T21)A(T32)A(T43)A(T54)A(T65)A(T76)AB chain kinematics: (T70)B=(T10)B(T21)B(T32)B(T43)B(T54)B(T65)B(T76)BCchain kinematics: (T70)C=(T10)C(T21)C(T32)C(T43)C(T54)C(T65)C(T76)C

Thus, the inverse kinematics of the system can be obtained by the link parameters and each chain kinematics as follows:

(3) $$\begin{array}{l} hA=b+Pz7+L\text{sin}(\theta_{A3})\text{cos}(\theta_{A4})\\ hB=b+Pz7+L\text{sin}(\theta_{B3})\text{cos}(\theta_{B4})\\ hC=b+Pz7+L\text{sin}(\theta_{C3})\text{cos}(\theta_{C4}) \end{array}$$

where:

$$\begin{array}{c} \theta_{A3}={\text{cos}}^{-1}\left(\frac{R-r-a-\frac{\sqrt{3}}{2}\times Px7+\frac{1}{2}\times Py7}{L\times \text{cos}(\theta_{A4})}\right),\;\theta_{B3}={\text{cos}}^{-1}\left(\frac{R-r-a+\frac{\sqrt{3}}{2}\times Px7+\frac{1}{2}\times Py7}{L\times \text{cos}(\theta_{B4})}\right)\\ \theta_{C3}={\text{cos}}^{-1}\left(\frac{R-r-a-Py7}{L\times \text{cos}(\theta_{C4})}\right),\;\theta_{A4}={\text{sin}}^{-1}\left(\frac{-Px7-\sqrt{3}\times Py7}{2\times L}\right),\;\theta_{B4}={\text{sin}}^{-1}\left(\frac{-Px7+\sqrt{3}\times Py7}{2\times L}\right)\\ \theta_{C4}={\text{sin}}^{-1}\left(\frac{Px7}{L}\right) \end{array}$$

where *hA*, *hB*, *hC* are the position of the joint along *A*, *B*, *C* rod-less pneumatic cylinders; *b* is the distance between the end-effector and the centroid of the load; (*Px*7, *Py*7, *Pz*7) is the pose of the end-effector; *L* is the length of the parallel link; *θ*_*A*3_, *θ*_*B*3_ and *θ*_*C*3_ are the joint angle of *P*3 on each chain, *θ*_*A*4_, *θ*_*B*4_ and *θ*_*C*4_ are the joint angles of *P*4 on each chain; *R* is the distance between the centroid of the bottom plate and each rod-less pneumatic cylinder; *r* is the distance between the centroid of the load and the *P*5 joint; *a* is the width of the slide on the rod-less pneumatic cylinder.

#### 3.1.2. Forward Kinematics

The forward kinematics is to compute the pose of the end-effector of the moving platform from the given input of the three linear actuators. Therefore, by the inverse kinematics, the mathematic models of the forward kinematics can be described as follows:

(4) $$\begin{array}{c} Px7=L\text{sin}(\theta_{C4})\\ Py7=R-r-a-L\text{cos}(\theta_{C4})\text{cos}(\theta_{C3})\\ Pz7=hC-b-L\text{sin}(\theta_{C3})\text{cos}(\theta_{C4}) \end{array}$$

where the unknown parameters are the location of the end-effector *P*7 = [*Px*7, *Py*7, *Pz*7] to be determined from the given joint angles.

### 3.2. Control Strategy

In the rodless pneumatic servo system, the opening area of servo valve’s orifice depends on the control input to affect the air flow. As the air flows into the rodless pneumatic cylinder, the pressure difference between two cylinder chambers is caused and results in the motion of the pneumatic cylinder. In order to analyze the rodless pneumatic servo system, dynamic models of the servo system are derived. The dynamic models of the pneumatic servo system primarily comprise the dynamics of the pneumatic servo valve, the mass flow rate of the pneumatic servo valve, the continuity equation and the motion equation. The state equations of the pneumatic servo system are derived as follows:

(5) $$\begin{array}{l} {\dot{x}}_{1}(t)=x_{2}(t)\\ {\dot{x}}_{2}(t)=\frac{\left(Ax_{3}(t)-Ax_{4}(t))\text{sgn}(x_{1}(t))-K_{f}x_{2}(t)-K_{S-c}(x_{1}(t))S(x_{2}(t),x_{3}(t),x_{4}(t))-\mathrm{Mg}\text{sgn}(x_{1}(t)\right)}{M}\\ {\dot{x}}_{3}(t)=\frac{-kx_{2}(t)x_{3}(t)}{x_{1}(t)+\Delta }+\frac{{kRT}_{s}C_{d}C_{0}\mathrm{wu}(t)\hat{f}(x_{3}(t),P_{s}(t),P_{e}(t))}{A(x_{1}(t)+\Delta )}\\ {\dot{x}}_{4}(t)=\frac{kx_{2}(t)x_{4}(t)}{l-x_{1}(t)+\Delta }+\frac{{kRT}_{s}C_{d}C_{0}\mathrm{wu}(t)\hat{f}(x_{4}(t),P_{s}(t),P_{e}(t))}{A(l-x_{1}(t)+\Delta )} \end{array}$$

where *A* is piston area; *P_e_* = 1 × 10^5^ *N/m*^2^ is exhaust pressure; *C_d_* = 0.8 is discharge coefficient; *P_s_* = 1 × 10^5^ *N/m*^2^ is supply pressure; *C_0_* is flow constant; *T_s_* = 293 *K* is cylinder air temperature; Δ is the general residual chamber volume, *k* = 1.4 is specific heat constant; *K_f_* is viscous frictional coefficient; *V* is volume; *P_atm_* is atmospheric pressure; *w* is port width; *l* is stroke and *x* ∈ [0,*l*], *M* is payload; *P_r_* is ratio between down- and up-stream pressure; *P_u_* is up-stream pressure; *P_d_* is down-stream pressure; *R*=287 *J*/(*kg·K*) is universal gas constant. In order to develop the path controller for the three pneumatic servo actuators, Fourier series-based adaptive sliding-mode controller with *H_∞_* tracking performance (FSB-ASMC+*H_∞_*) [8] is used in this study to solve the high non-linearity and time-varying problems. The FSB-ASMC+*H_∞_* controller contains the Fourier series-based adaptive sliding mode controller and *H_∞_* tracking performance design technique. The Fourier series-based adaptive sliding mode controller can handle the high non-linear and time-varying problems. Furthermore, the *H_∞_* tracking performance design technique is to overcome the function approximation errors, un-modeled dynamics, disturbances, and to reduce the chattering effect caused by the sliding mode control. A general nonlinear system is shown as follows:

(6) $$y{\left(t\right)}^{\left(n\right)}=F\left(x,t\right)+g\left(x,t\right)u\left(t\right)$$

where *y*(*t*) is the output of the system, *F*(**x**, *t*) and *g*(**x**, *t*) are unknown time-varying function, and *u*(*t*) is the control input of the system. The functional approximation technique to approximate the functions *F*(**x**, *t*) and *g*(**x**, *t*), and Equation (7) can be rewritten as [8]:

(7) $$y{\left(t\right)}^{\left(n\right)}=\left(W_{F}^{T}q_{F}\left(t\right)+\varepsilon_{F}\left(t\right)\right)+\left(W_{g}^{T}q_{g}\left(t\right)+\varepsilon_{g}\left(t\right)\right)u\left(t\right)$$

where $W_{F}^{T}q_{F}\left(t\right)$ and $W_{g}^{T}q_{g}\left(t\right)$ are approximations to the uncertain time-varying functions *F*(**x**, *t*) and *g*(**x**, *t*); ε*_F_*(*t*) and ε*_g_*(*t*) are the truncation errors of the approximations of *F*(**x**, *t*) and *g*(**x**, *t*). Define the output error as:

(8) $$e(t)=y(t)-y_{m}(t)$$

where *y_m_*(*t*) is a given bounded reference signal. The sliding surface is described as:

(9) $$s=a_{1}e(t)+a_{2}\dot{e}(t)+\ldots +e^{\left(n-1\right)}(t)$$

where *a_i_* are chosen such that $\sum_{i=1}^{n}a_{i}\lambda^{i-1}$ is a Hurwitz polynomial. In addition, the *H_∞_* tracking performance design technique is proposed to reduce the chattering effect affected by sliding control. Therefore, the control input is chosen as [8]:

(10) $$u(t)=\frac{-{\hat{W}}_{F}^{T}q_{F}(t)-\sum_{i=1}^{n-1}a_{i}e_{i+1}(t)-\sum_{i=1}^{n-1}p_{(n-1)i}e_{i}(t)+y_{m}^{\left(n\right)}(t)-\frac{s}{2\rho^{2}}}{{\hat{W}}_{g}^{T}q_{g}(t)}$$

where ${\hat{W}}_{F}^{T}$ and ${\hat{W}}_{g}^{T}$ are the estimations of $W_{F}^{T}$ and $W_{g}^{T}$; *p*_(*n*–1)*i*_ denotes elements of **P** satisfying the Lyapunov matrix equation $A_{1}^{T}P+PA_{1}=-Q$, and *ρ* > 0 is the design constant for attenuation level.

### 3.3. Controller Design

**Figure 4.**
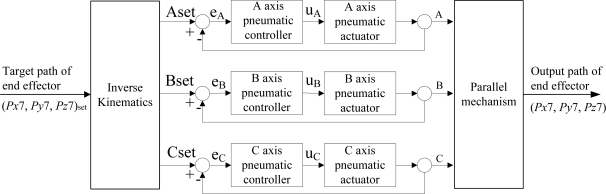
control block diagram for the complex 3D parallel mechanism robot.

In this study, the control input is chosen as:

(11) $$u(t)=\frac{-{\hat{W}}_{F}^{T}q_{F}(t)-a_{1}\dot{e}-a_{2}\ddot{e}-p_{21}e-p_{22}\dot{e}+y_{m}^{\left(3\right)}(t)-\frac{s}{2\rho^{2}}}{{\hat{W}}_{g}^{T}q_{g}(t)}$$

where *s* = *a*_1_*e* + *a*_2_*ė* + *ë* and *e* is position error. The initial values of Fourier coefficients **Ŵ***_F_* and **Ŵ***_G_* are [0, 0, · · ·,0]_1×(2×5+1)_ and [20000, 0, · · ·,0]_1×(2×5+1)_; and $Q=\left[\begin{array}{cc} 1 & 2\\ 2 & 5 \end{array}\right]$. The other controller parameters are shown in Table 3. Furthermore, Figure 4 shows the control block diagram for the complex 3D parallel mechanism robot.

## 4. Experiments

The objective of this paper was to develop the path tracking control of the end-effector of the complex 3D parallel mechanism robot driven by the three vertical pneumatic servo actuators. In order to confirm the feasibility, the experiments of path tracking control for the end-effector in different tracking trajectories are implemented in the developed full-scale test rig of the 3D pneumatic parallel mechanism robot respectively. Two different 3D desired path trajectories for the end-effector, such as a circle path trajectory and a ball path trajectory, are presented in the experiments. Finally, the sensor collaboration strategy combining three position sensors of pneumatic actuators and the stereo vision system is implemented to on-line compensate the position error due to the tolerance of assembly and manufacture of joints and chains.

### 4.1. Path Tracking Control of Circle Trajectory

**Figure 5.**
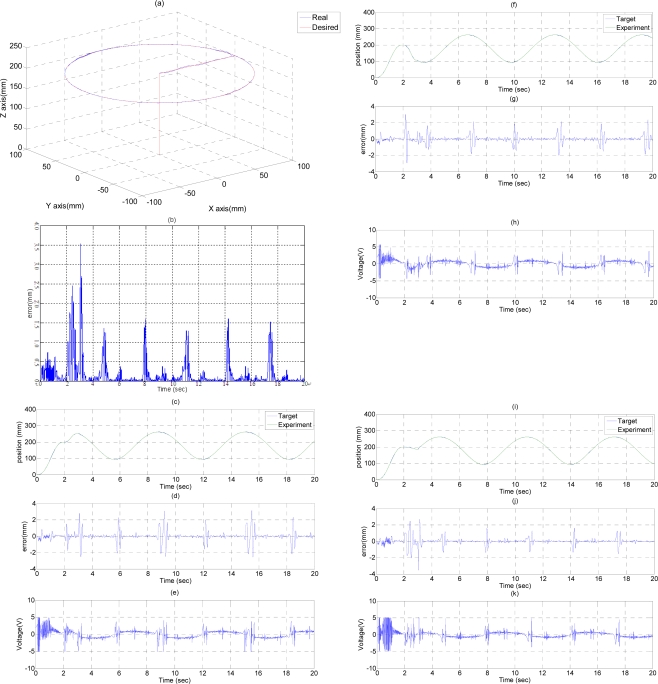
Experimental results of path tracking control in a circle trajectory of end-effector: (**a**) system response of an end-effector; (**b**) tracking error of an end-effector; (**c**) system response of A-axis; (**d**) tracking error of A-axis; (**e**) control signal of A-axis; (**f**) system response of B-axis; (**g**) tracking error of B-axis; (**h**) control signal of B-axis; (**i**) system response of C-axis; (**j**) tracking error of C-axis; (**k**) control signal of C-axis.

In the experiment circle trajectory path tracking control, the end-effector moves along a circle trajectory with a specific diameter on a horizontal plane for path tracking control. Figure 5 shows the experimental results of the path tracking control for the end-effector of the 3D pneumatic parallel mechanism robot in a circle trajectory controlled by FSB-ASMC+H_∞_. The end-effector firstly moves along the Z-axis from (X, Y, Z) = (0, 0, 0) to (X, Y, Z) = (0, 0, 200) in 2 s, and then moves alone the X-axis from (X, Y, Z) = (0, 0, 200) to (X, Y, Z) = (100, 0, 200) in 1 s. Finally, the end-effector moves along a circle trajectory with center of (X, Y, Z) = (100, 0, 200) and a diameter of 200 mm in 17 s. The desired path and the system response of the end-effector in the circle trajectory are shown in Figure 5(a). Figure 5(b) shows the tracking errors of the end-effector in the circle trajectory, where the maximum tracking error, about 3.5 mm at 3 s, results from the different motion path change. Except this point, the tracking error during the circle trajectory can be kept within 1.6 mm. Figures 5(c,f,i) show the desired paths calculated by the inverse kinematics and the system response for the three vertical pneumatic servo actuators in the circle trajectory respectively. Figures 5(d,g,j) show the tracking errors for the A-, B- and C-axial vertical pneumatic servo actuators in the circle trajectory separately, where the maximum tracking errors, about 3.5 mm at 3 s, occurs in the different path change point. Except that, the path tracking error of each vertical pneumatic axis can be controlled within 3 mm, 2 mm, and 1.7 mm for A-, B- and C-axis, which mainly results from the friction force at the moment of motion direction change. Figures 5(e,h,k) show the control signals of the three vertical pneumatic servo actuators in the circle trajectory. Therefore, the desired tracking performance of the end-effector in a circle trajectory can be achieved satisfactorily.

### 4.2. Path Tracking Control of Ball Trajectory

**Figure 6.**
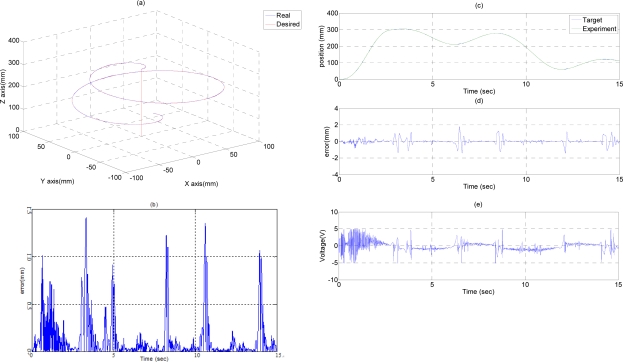

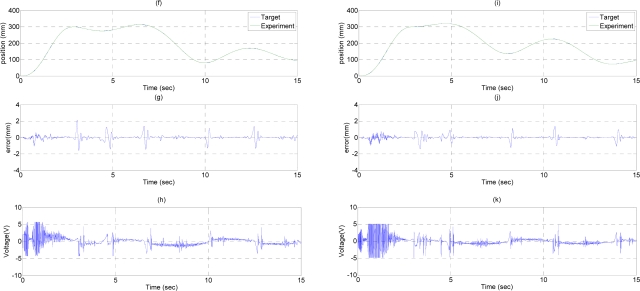
Experimental result of path tracking control in a ball trajectory: **(a)** system response of an end-effector; **(b)** tracking error of an end-effector; **(c)** system response of A-axis; **(d)** tracking error of A-axis; **(e)** control signal of A-axis; **(f)** system response of B-axis; **(g)** tracking error of B-axis; **(h)** control signal of B-axis; **(i)** system response of C-axis, **(j)** tracking error of C-axis; **(k)** control signal of C-axis.

In the ball trajectory path tracking control experiment, the end-effector moves along a ball trajectory with a specific diameter in the three-dimensional space. Figure 6 show the experimental results of the path tracking control for the end-effector in a ball trajectory controlled by FSB-AFSMC+H_∞_. The end-effector firstly moves from (X, Y, Z) = (0, 0, 0) to (X, Y, Z) = (0, 0, 300) in 3 s, and then moves along a ball trajectory with a diameter of 200 mm in 12 s. The desired path and the system response of the end-effector in the ball trajectory are shown in Figure 6(a). Figure 6(b) shows the tracking errors of the end-effector in the ball trajectory, where the maximum tracking errors can reach about 1.5 mm. Figures 6(c,f,i) show the desired paths calculated by the inverse kinematics and the system response of the A-, B- and C-axial vertical pneumatic actuators in the ball trajectory. Figures 6(d,g,j) show the tracking errors of the A-, B- and C-axial vertical pneumatic actuators in a ball trajectory, where the maximum tracking errors can reach about 1.9 mm, 2 mm and 1.6 mm for the A-, B- and C-axis owing to the influence of the friction force at the moment of motion direction change. Figures 6(e,h,k) show the control signals of the A-, B- and C-axis in the ball trajectory. Therefore, the desired tracking performance of the end-effector in a ball trajectory can be achieved adequately. Thus, the feasibility of the developed 3D parallel mechanism robot driven by three vertical pneumatic servo actuators are confirmed.

### 4.3. Improvement the Error Between the Calculated Position and the Stereo Vision Measuring Position of End-Effector Through Sensor Collaboration

In order to improve the accuracy of the calculated 3D position of the end-effector from the measuring results of the three position sensors, *i.e.*, optical linear encoders, of the pneumatic actuators through sensor collaboration, the stereo vision system which algorithm has been developed and described in detail in the authors’ previous publication [26] is used to measure the actual 3D position of the end-effector. An on-line sensor collaboration strategy is developed in this paper for the three position sensors and the stereo vision system to compensate the error between the calculating position and the stereo vision measuring position of the end-effector.

The desired position of the end-effector of the robot is (*x_d_*, *y_d_*, *z_d_*). The calculated position of the end-effector of the robot is (*x_c_*, *y_c_*, *z_c_*). The stereo vision measuring position of the end-effector of the robot is (*x_s_*, *y_s_*, *z_s_*). According to the experiment results in Figures 5 and 6, the control error between the desired position (*x_d_*, *y_d_*, *z_d_*) and the calculating position (*x_c_*, *y_c_*, *z_c_*) can be controlled within 2 mm range. Thus, the closed-loop control of the robot performs well, and the error between the stereo vision measuring position and the calculating position caused by the tolerance of joints and chains of the mechanism can be calculated. Therefore, the sensor collaboration strategy developed in this paper is to compensate this error between the stereo vision measuring position and the calculating position caused by the tolerance of joints and chains of the mechanism.

We define the compensation values of the end-effector of the robot as (Δ*x*, Δ*y*, Δ*z*), which is difference between the stereo vision measuring position (*x_s_*, *y_s_*, *z_s_*) and the calculating position (*x_c_*, *y_c_*, *z_c_*) of the end-effector:

(12) $$\left(\Delta x,\;\Delta y,\;\Delta z)=(x_{s},\;y_{s},\;z_{s})-(x_{c},\;y_{c},\;z_{c}\right)$$

Then, the compensation value (Δ*x*, Δ*y*, Δ*z*) is added to the desired position of the end-effector of the robot (*x_d_*, *y_d_*, *z_d_*) to achieve the new desired position of the end-effector (*x_d_n_*, *y_d_n_*, *z_d_n_*) for on-line compensating the error between the stereo vision measuring position and the calculating position caused by the tolerance of joints and chains of the mechanism:

(13) $$\left(x_{d\_n},\;y_{d\_n},\;z_{d\_n})=(x_{d},\;y_{d},\;z_{d})-(\Delta x,\;\Delta y,\;\Delta z\right)$$

Consequently, the new desired position of the end-effector (*x_d_n_*, *y_d_n_*, *z_d_n_*) is given to calculate the new desired position of the three vertical pneumatic actuators via inverse kinematics. Through the pneumatic servo control the new desired position of the three vertical pneumatic actuators can be achieved, and then the modified position of the end-effector can be reached.

**Figure 7.**
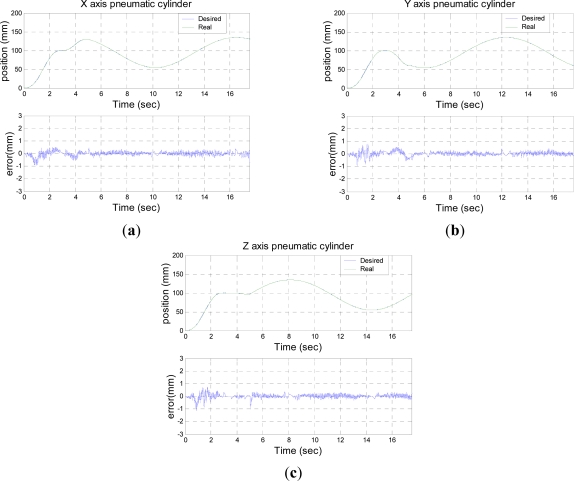
3D circle trajectory tracking result of three pneumatic cylinders measured by the three linear optical encoders; **(a)** position and error of *X*-axial pneumatic cylinder; **(b)** position and error of *Y*-axial pneumatic cylinder; **(c)** position and error of *Z*-axial pneumatic cylinder.

**Figure 8.**
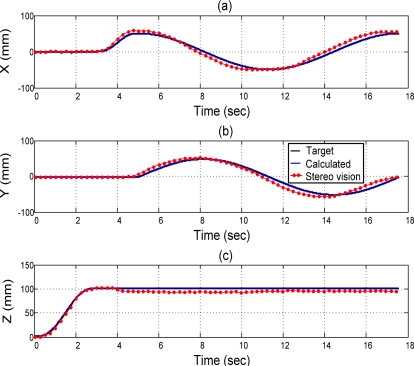
3D circle trajectory measurement result in 3D view of 3D circle trajectory of the end-effector by the stereo vision system in *X*, *Y*, and *Z*-axis, **(a)** in *X*-axis; **(b)** in *Y*-axis; and **(c)** in *Z*-axis.

**Figure 9.**
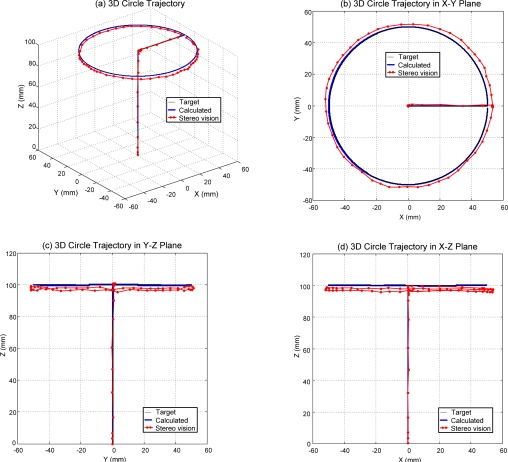
measurement results of the 3D circle trajectory of the end-effector by the stereo vision system in multiple views, **(a)** 3D circle trajectory measurement result in 3D view; **(b)** 3D circle trajectory measurement result in *X*–*Y* plane; **(c)** 3D circle trajectory measurement result in *Y*–*Z* plane; **(d)** 3D circle trajectory measurement result in *X*–*Z* plane.

Figures 7–9 shows the experimental results of 3D circle trajectory tracking control of the robot by the on-line sensor collaboration strategy. The desired trajectory of the end-effector in this section is planned to be a fifth order polynomial trajectory with stroke 100 mm at t ≤ 3 s in the *Z*-direction. At 3 s ≤ *t* ≤ 5 s, a fifth order polynomial trajectory with stroke 50 mm is planned in the *X*-direction. Finally, a 100 mm constant position in *Z*-direction, a cosine trajectory in *X*-direction and a sine trajectory in *Y*-direction both with an amplitude of 50 mm and a frequency of 0.5 rad/s are planned at 5 s ≤ t ≤ 17.5 s for achieving a circular motion in the *X*–*Y* plane.

Figure 7 shows the desired trajectory of three axial pneumatic cylinders which is generated by the inverse kinematics, the position response measured by the three linear optical encoders and the control error of trajectory tracking of each pneumatic cylinder.

Figure 8 shows the measurement results in 3D view of a 3D circle trajectory of the end-effector by the stereo vision system in *X*, *Y*, and *Z*-axis, including the *X*-axis in Figure 8(a), the *Y*-axis in Figure 8(b), and the *Z*-axis in Figure 8(c), respectively.

Figure 9 shows the measuring results of the 3D circle trajectory of the end-effector by the stereo vision system in multiple views, such as 3D circle trajectory measuring result in 3D view in Figure 9(a), 3D circle trajectory measurement result in *X*–*Y* plane in Figure 9(b), 3D circle trajectory measurement result in the *Y*–*Z* plane in Figure 9(c), and the 3D circle trajectory measurement result in the *X*–*Z* plane in Figure 9(d). According to the experimental results of the sensor collaboration, the maximum difference of radius of the circle is close to 5 mm. An approximate 3 mm excess upward movement in the *Z*-direction of the end-effector exists during the *X*-direction trajectory planned at 3 s ≤ *t* ≤ 5 s. An approximate 5 mm excess movement in the *X*-direction of the end-effector exists during the the *X*-direction trajectory planned at 3 s ≤ *t* ≤ 5 s. The variation of the *Z* component during the circle trajectory at 5 s ≤ *t* ≤ 17.5 s to within 5 mm can be perceived in both Figure 9(c) and Figure 9(d). Because the sampling frequency of pneumatic servo control of robot is 900 Hz and the frame rate of the stereo vision system is 30 Hz, the on-line sensor collaboration strategy is executed with 30 Hz. That is, the compensation value (Δ*x*, Δ*y*, Δ*z*) of the sensor collaboration strategy is calculated every 30 sampling points and this compensation value is kept in the 30 sampling points. The frame rate of the stereo vision system limited by the image acquisition card restricts the effect of the sensor collaboration strategy. The improvement of the error due to the tolerance of assembly and manufacture of joints and chains by the sensor collaboration strategy is confirmed experimentally. The effect can be further increased by using an image acquisition card with higher frame rate.

## 5. Conclusions

In this study, a novel 3D parallel mechanism robot driven by three vertical pneumatic servo actuators combined with a stereo vision system was developed and implemented experimentally for complex 3D path tracking control in a full-scale test rig. In order to analyze the parallel mechanism of the robot, D-H notation is used to resolve the kinematics problem of the robot. For realizing the path tracking control, FSB-ASMC+H_∞_ controller was developed to overcome the nonlinearities, and the time-varying problems of the pneumatic servo system. In order to verify the proposed robot system experimentally, a full-scale test rig of the complex 3D parallel mechanism robot was set up. To further confirm the feasibility of the proposed system, different complex 3D trajectories of the end-effector of the robot, including a circle trajectory and a ball trajectory, were implemented successfully in the experiment of the test rig. Through sensor collaboration of the three position sensors of pneumatic actuators and the stereo vision system, the calculated position of the end-effector by the three position sensors of pneumatic actuators through kinematics can be compared and calibrated by the stereo vision system which was implemented to measure the actual position of the end-effector. Finally, the experimental results clarify that the complex 3D parallel mechanism robot driven by three vertical pneumatic servo actuators can perform successfully for complex 3D path tracking control. Through the calibration by the stereo vision system, the measurement errors of the calculated position of the end-effector can be considered. In addition, the proposed parallel mechanism robot combined with the vision measurement could be applied in the fields of flat panel display glass substrate conveyors and solar cell industries in the future.

## Figures and Tables

**Table 1.** Specifications of the test rig.

**Components**	**Specifications**
Pneumatic rodless cylinder	Diameter: 25 mm; Stroke: 500 mm
Proportional servo valve	Valve function: 5/3-way; Input: 0∼10 V
Optical encoder	Range: 500 mm; Resolution: 0.1 μm
AD/DA cards	12 bit A/D×16 CH; 12 bit D/A×6 CH; D/I, D/O×16 CH
Stereo vision system	Image sensor: Interline CCD Video output pixels: 648(H) × 492(V)(Under non-interlace) Scanning area: 1/3; Scanning lines: 525 lines Interlace: 1/60 s Non-interlace mode; 1/120 s 2:1 Interlace mode
Image acquisition card	Available formats: RS-170/NTSC 30 frames/s interlaced; CCIR/PAL 25 frames/s interlaced; VGA 60 Hz, 640 × 480 resolution Resolution: 8 or 10 bits; Sampling rate: 2 M∼40 MHz

**Table 2.** Link parameters of the complex 3D robot.

	**A Chain**				**B Chain**				**C Chain**			
	*a*_*i*–1_	*α*_*i*–1_	*θ*_*i*_	*d*_*i*_	*a*_*i*–1_	*α*_*i*–1_	*θ_i_*	*d*_*i*_	*a*_*i*–1_	*α*_*i*–1_	*θ_i_*	*d_i_*
*P*_0_ ⇒ *P*_1_	−*R*	150°	0°	0	−*R*	30°	0°	0	−*R*	−90°	0°	0
*P*_1_ ⇒ *P*_2_	*a*	0^0^	−90°	*hA*	*a*	0°	−90°	*hB*	*a*	0°	−90°	*hC*
*P*_2_ ⇒ *P*_3_	0	*θ*_*A*3_	90°	0	0	*θ*_*B*3_	90°	0	0	*θ*_*C*3_	90°	0
*P*_3_ ⇒ *P*_4_	*L*	*θ*_*A*4_	180°	0	*L*	*θ*_*B*4_	−180°	0	*L*	*θ*_*C*4_	180°	0
*P*_4_ ⇒ *P*_5_	0	*θ*_*A*5_	−90°	0	0	*θ*_*B*5_	−90°	0	0	*θ*_*C*5_	−90°	0
*P*_5_ ⇒ *P*_6_	*r*	*θ*_*A*6_	−90°	0	*r*	*θ*_*B*6_	−90°	0	*r*	*θ*_*C*6_	−90°	0
*P*_6_ ⇒ *P*_7_	0	−150°	0°	−*b*	0	−30°	0°	−*b*	0	90°	0°	−*b*

**Table 3.** Controller parameters of the servo pneumatic system for path tracking control.

	*a*_1_	*a*_2_	*ρ*	Γ_1_	Γ_2_

The value of control parameter	40	5	0.2	83×*I*_11×11_	1.25×10^−4^×*I*_11×11_
